# News and Items

**Published:** 1868-12-01

**Authors:** 


*News and Items.*

Dr. Lockhart having resigned the Superintendency of the Indiana Insane Asylum, the Board of Trustees have elected Dr. Orpheus Everts, of Michigan City, to the position. An appointment eminently "fit to be made."

S. E. Scanland, M.D., a well known graduate of "Rush," has drawn a first-class prize in the great lottery of of life, having captured and led to the hymeneal altar, the beautiful and accomplished Miss Agnes Leonard, well known to the literary world as "Mollie Myrtle." Rev. D. P. Henderson officiated. The very best wishes of The Journal attend them.

The entertainment given by the profession and citizens of Philadelphia, to Professors Gross and Pancoast, on their return from Europe, proved an eminent success, just as it should have been. We shall give an extract from the beautiful address of Prof. Gross in the next number.

The latest editions of Flint's Practice of Medicine, etc.; the second volume of Aitken's Practice, are respectively on file for speedy notice.

The New York Hospital is to be torn down, and the grounds leased for $140,000 annually. The Hospital will be reërected at Bloomingdale.

The policy is announced that hereafter surgical and medical appointments in the army, navy, and civil service of the government will be made only as subservient to political considerations. The notice seems to be a work of sepererogation.

W. Alphonso Wood, M.D., a highly respected practitioner of medicine, is reported to have died recently and suddenly. The notice sent to the Journal is authenticated by no name or date. When they are sent us we shall publish the resolutions said to have been passed by a local society on the occasion. Dr. Wood was one of nature's noblemen, still further ennobled by professional and civic culture.

